# Prevalence of COVID-19 vaccine acceptance among migrant and refugee groups: A systematic review and *meta*-analysis

**DOI:** 10.1016/j.jvacx.2023.100308

**Published:** 2023-05-06

**Authors:** Zainab Alimoradi, Malik Sallam, Elahe Jafari, Marc N. Potenza, Amir H. Pakpour

**Affiliations:** a Social Determinants of Health Research Center, Research Institute for Prevention of Non-Communicable Diseases, Qazvin University of Medical Sciences, Qazvin, Iran; b Department of Pathology, Microbiology and Forensic Medicine, School of Medicine, The University of Jordan, Amman, Jordan; c Department of Clinical Laboratories and Forensic Medicine, Jordan University Hospital, Amman, Jordan; d Department of Translational Medicine, Faculty of Medicine, Lund University, Malmö, Sweden; e Departments of Psychiatry and Neuroscience and the Child Study Center and Wu Tsai Institute, Yale School of Medicine / Yale University, New Haven, CT, USA; f Connecticut Mental Health Center, New Haven, CT, USA; g Connecticut Council on Problem Gambling, Wethersfield, CT, USA; h Department of Nursing, School of Health and Welfare, Jönköping University, Jönköping, Sweden

**Keywords:** Minority, Immigration, Acceptance, COVID-19 vaccination

## Abstract

**Objectives:**

Understanding COVID-19 vaccine hesitancy among migrant and refugee groups is critical for achieving vaccine equity. Therefore, we aimed to estimate the prevalence of COVID-19 vaccine acceptance among migrant and refugee populations.

**Methods:**

A systematic review (PROSPERO: CRD42022333337) was conducted (December 2019–July 2022) using PubMed, Scopus, Web of Science, ProQuest and Google Scholar.

**Results:**

Nineteen studies from 12 countries were included. The pooled estimated prevalence of COVID-19 vaccine willingness among migrant and refugee groups was 70% (19 studies, 95% CI: 62.3–77.4%, I^2^: 99.19%, τ^2^: 0.03). Female and male participants did not differ significantly with each other (*p* = 0.64). Although no individual variable contributed statistically significantly in multivariable *meta*-regression analysis, the multivariable model that considered methodological quality, mean age of participants, participant group and country of origin explained 67% of variance.

**Discussion:**

Proportions of migrant/refugee groups receiving COVID-19 vaccinations approximated those observed among general populations. Additional studies are needed to examine factors relating to vaccine willingness to identify the most significant factors that may be targeted in interventions.

## 1 Introduction

Since the declaration of coronavirus disease 2019 (COVID-19) as a pandemic, the devastating health impact has involved more than 6.4 million deaths worldwide and more than 590 million confirmed cases as of August 19, 2022 [1]. Due to the limited availability of antiviral treatment options, vaccinations against COVID-19 may be considered as the mainstay preventive measure [2], [3]. However, the utility of COVID-19 vaccination can be hampered by several factors including: (1) the repeated emergence of severe acute respiratory syndrome coronavirus 2 (SARS-CoV-2) variants with immune-system-escaping properties [4], [5]; (2) the limited universal vaccination coverage linked to unequal vaccine supplies [6], [7]; and, (3) the phenomenon of vaccination hesitancy, which is widely prevalent globally, with common occurrence in the Middle East, North Africa, Europe, Central Asia, and Western/Central Africa [8], [9].

Vaccination requires the inoculation of a certain proportion of the at-risk population in order to achieve immunity of the whole population [10]. Despite being recognized as one of the most successful public health measures, many people prefer not to be vaccinated, citing safety concerns and questioning the necessity of vaccination [10], [11], [12].

Based on the latest World Health Organization (WHO) report, almost one billion people in lower-income countries remained unvaccinated as of May 22, 2022 [13]. Only 57 countries have vaccinated 70% of their population, and almost all were high-income countries [13]. Supporting vaccination in all countries to reach 70% vaccination is necessary, including targeting: 100% of those aged over 60 years; 100% of healthcare workers; and 100% of those with underlying comorbidities [13]. Published reports from several countries globally have explored attitudes towards COVID-19 vaccination [14], [15], [16], [17], [18], [19], [20]. The results of these studies have shown extensive variability in COVID-19 vaccine acceptance, with high rates of vaccine hesitancy in several studied populations [8], [9]. Among the population subgroups with lower COVID-19 vaccination rates are migrant and refugee groups [21], [22].

Paying attention to migrant and refugee populations’ health is a very important issue [23]. At the 72nd World Health Assembly in 2019, the WHO prioritized the health of refugee and migrant groups and recognized that access to health care services, including vaccinations, is more difficult for such people on the move [24], [25]. In addition, human displacement is often associated with transmission of infectious diseases [26]. Although vaccination is often required for migration and refugee resettlement, many immigrant communities experience lower immunization rates and a higher burden of vaccine-preventable diseases (VPDs) than host populations [27], [28]. When migration is forced (refugee) and large-scale, migration can be very disruptive to both the refugee population and the host country, weakening the resilience of the health system and jeopardizing the provision of health services, including vaccination services [26], [29].

Among immigrant groups, some concerns about vaccination may be rooted in the home country's culture or experience, and thus concerns may exist prior to immigration [30]. Racism (real or perceived) in the host country may make some immigrant populations reluctant to integrate and vaccinate [31], [32]. Finally, the prevalence of VPDs among some immigrant communities in host countries with high vaccination coverage suggests that vaccine hesitancy may be a factor in their health vulnerability [33], [34]. The achievement of vaccine equity is critical for proper control of infectious diseases, including the restriction of COVID-19 spread [35], [36]. Thus, special attention should be given to vulnerable and underprivileged groups including migrant and refugee populations [37]. These groups are often challenged by several barriers precluding the appropriate attainment of their health needs [38]. The difficulties experienced by migrant or refugee groups include overcrowding, poverty, poor health literacy besides the restricted access to healthcare systems due to language and cultural barriers [39], [40], [41], [42]. Additionally, refugee groups are prone to poor mental health given frequent war exposure and persecution trauma [43]. These experiences may have been exacerbated during the COVID-19 pandemic; therefore, the WHO advocated the need to focus on these disadvantaged groups [44]. Specific exacerbations relate to high risk for SARS-CoV-2 acquisition and severe COVID-19-related morbidity and mortality within migrant and refugee groups [45], [46].

The primary goal of this study was to estimate the prevalence of the acceptance or willingness to receive COVID-19 vaccination in migrant and refugee populations. Secondary objectives included examining the factors influencing the acceptance of this vaccine, examining the acceptance of the vaccine in different population subgroups, evaluating studies and evaluating gender-related differences in COVID-19 vaccine acceptance.

## 2 Methods

### 2.1 Protocol and registration

The study protocol was registered in PROSPERO, International prospective register of systematic reviews under decree code of CRD42022333337 [47]. This systematic review was conducted based on the Preferred Reporting Items for Systematic Reviews and Meta-Analyses (PRISMA) guidelines [48].

### 2.2 Eligibility criteria

The eligibility criteria were defined based on population, exposure, comparator and outcome (PECO) components [49]. The PECO framework is a well-defined approach to formulate search questions assessing associations between exposures and outcomes, including in various fields of health.

Considering PECO components in the current systematic review, the eligibility criteria were set as follows: (1) Population: migrant and refugee groups with no limitation regarding their demographic characteristics; (2) Exposure: COVID-19 pandemic; (3) Comparison: populations other than migrant and refugee groups; (4) Outcome: Frequency or prevalence of COVID-19 vaccination acceptance (and/or no hesitance) or willingness to receive COVID-19 vaccines; and (5) Study design: observational studies, published between December 2019 and July 2022, using English language, peer-reviewed and published papers, reporting data on frequency or prevalence of COVID-19 vaccination among migrant and refugee groups.

Migrant people in this study were considered based on The International Organization for Migration (IOM) definition as follows: “Any person who is on the move or has moved across an international border or in a country away from his or her usual place of residence, regardless of (1) the legal status of the person; (2) whether the movement is voluntary or involuntary; (3) what are the causes of motion; or (4) how long is the stay” [50], [51]. Additionally, studies that investigated COVID-19 vaccine acceptance among refugee populations were also included.

### 2.3 Information sources

Academic databases including PubMed, Scopus, Web of Science (WoS), and ProQuest were systematically searched from the start of December 2019 to the end of July 2022. To have a more comprehensive search, grey literature including Google Scholar and references lists of the included publications were independently searched.

### 2.4 Search strategy

The main search terms included COVID-19, vaccine and migrant groups. The core search strategy was (“COVID 19 Vaccines” OR (Vaccines AND “COVID-19”) OR “COVID-19 Virus Vaccines” OR “COVID 19 Virus Vaccines” OR (Vaccines AND “COVID-19 Virus”) OR (“Virus Vaccines” AND COVID-19) OR “COVID-19 Virus Vaccine” OR “COVID 19 Virus Vaccine” OR (Vaccine AND “COVID-19 Virus”) OR (Virus Vaccine AND COVID-19) OR “COVID19 Virus Vaccines” OR (Vaccines AND “COVID19 Virus”) OR (“Virus Vaccines” AND COVID19) OR “COVID19 Virus Vaccine” OR (Vaccine AND “COVID19 Virus”) OR (Virus Vaccine AND COVID19) OR “COVID19 Vaccines” OR (Vaccines AND COVID19) OR “COVID19 Vaccine” OR (Vaccine AND COVID19) OR “SARS-CoV-2 Vaccines” OR “SARS CoV 2 Vaccines” OR (Vaccines AND SARS-CoV-2) OR “SARS-CoV-2 Vaccine” OR “SARS CoV 2 Vaccine” OR (Vaccine AND SARS-CoV-2) OR “SARS2 Vaccines” OR (Vaccines AND SARS2) OR “SARS2 Vaccine” OR (Vaccine AND SARS2) OR “Coronavirus Disease 2019 Vaccines” OR “Coronavirus Disease 2019 Vaccine” OR “Coronavirus Disease 2019 Virus Vaccine” OR “Coronavirus Disease 2019 Virus Vaccines” OR “Coronavirus Disease-19 Vaccines” OR “Coronavirus Disease 19 Vaccines” OR (Vaccines AND “Coronavirus Disease-19”) OR “Coronavirus Disease-19 Vaccine” OR “Coronavirus Disease 19 Vaccine” OR (Vaccine AND “Coronavirus Disease-19”) OR “COVID 19 Vaccine” OR (Vaccine AND COVID 19) OR “2019-nCoV Vaccine” OR “2019 nCoV Vaccine” OR (Vaccine AND 2019-nCoV) OR “2019 Novel Coronavirus Vaccines” OR “2019 Novel Coronavirus Vaccine” OR “2019-nCoV Vaccines” OR “2019 nCoV Vaccines” OR (Vaccines AND 2019-nCoV) OR “COVID-19 Vaccine” OR (Vaccine AND COVID-19) OR “SARS Coronavirus 2 Vaccines”) AND ((“Transients and Migrants”) OR emigrant* OR Immigrant* OR Foreigner* OR refugee*). The search syntax was adapted for each database based on their advanced search attributes.

### 2.5 Study selection

First, titles and abstracts of all retrieved papers during the electronic and manual search processes were evaluated based on the inclusion criteria. This was followed by examination of the full texts of the potentially relevant articles based on the above-mentioned criteria. These processes were performed independently by two reviewers (Z.A. and A.H.P). Initial disagreements about the selection of studies were resolved through discussions.

### 2.6 Data collection process and data items

Data were extracted and recorded in pre-designed Excel datasheets by two reviewers independently. The following data were abstracted from each study: first-author name; country in which the study had been conducted; sample size; year(s) of data collection; participants' ages; educational status; geographical location; type of study; quality of study; and raw data to calculate prevalence of participants’ willingness to be vaccinated.

### 2.7 Risk of bias in individual studies

The Newcastle–Ottawa Scale (NOS) was used to assess risk of bias within included studies. This checklist evaluates the methodological quality of observational studies in the following three sections: selection, comparability, and outcome [52], [53]. The maximal acquirable score on the NOS checklist is 9 for each study. Studies with less than five points were classified as having a high risk of bias [53]. Methodological quality status was not considered as an eligibility criterion. However, the effect of methodological quality on the pooled effect size was assessed in the subgroup analysis and *meta*-regression.

### 2.8 Summary measures

Two summary measures were determined based on study objectives: (1) frequency or prevalence of the acceptance of the COVID-19 vaccine and their 95% confidence intervals (CIs); (2) contributing factors influencing acceptance of COVID-19 vaccines.

### 2.9 Synthesis of results

Numerical evidence regarding the prevalence of COVID-19 vaccine acceptance was quantitatively synthesized using STATA software version 14. Meta-analysis using a random-effects model was conducted to consider both within-study and between-study variances [54]. Severity of heterogeneity was estimated using the I^2^ index [55]. The prevalence of COVID-19 vaccine acceptance and its 95% CI was the selected key measure for the first objective of present study. For the second objective of the present study, a narrative synthesis was used due to methodological heterogeneity of variables and measures.

### 2.10 Risk of bias across studies

Funnel plot and Begg's test were used to assess publication bias [56]. Meta-trim with the fill and trim method was used to correct probable publication bias [57]. The Jackknife method was used for sensitivity analysis and probable single study effect on pooled effect size [58].

### 2.11 Additional analyses

To investigate predictor variables for COVID-19 vaccine acceptance, *meta*-regression was conducted. Univariable *meta*-regression was used to assess moderators of vaccine acceptance.

## 3 Results

### 3.1 Study screening and selection process

**Fig. 1:**
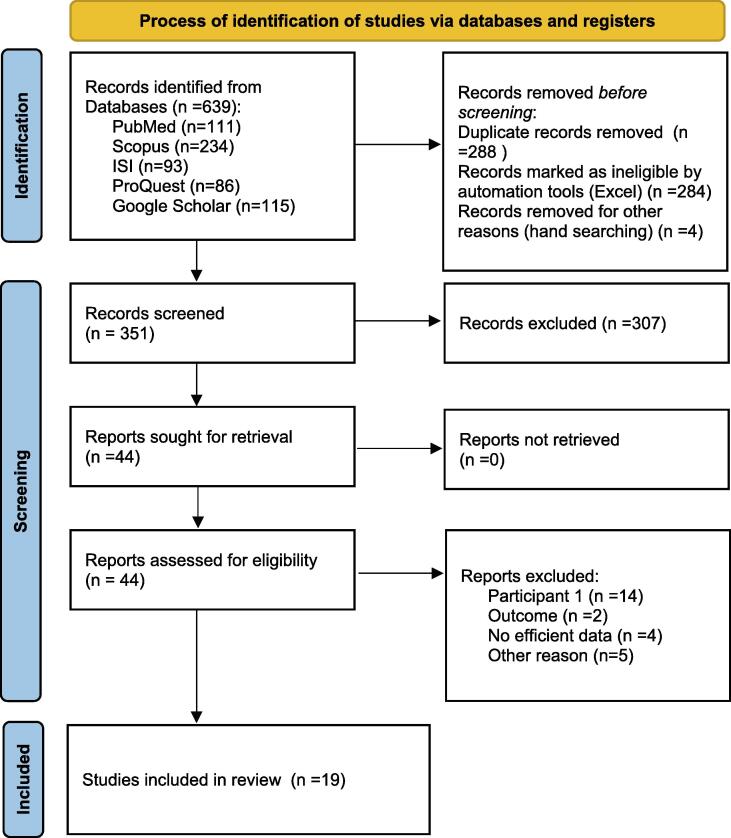
The search process based on the PRISMA flowchart.

The initial search in four academic databases as well as Google Scholar resulted in retrieval of 639 records: PubMed (n = 111); Scopus (n = 234); WoS (n = 93); ProQuest (n = 86); and Google Scholar (n = 115). After removing duplicates (n = 288), the remaining manuscripts were screened based on their titles and abstracts. Finally, 351 papers appeared to be potentially eligible and their full-texts were reviewed. In this process, 19 studies met the eligibility criteria, and 17 studies were pooled in the *meta*-analysis. The search process based on the PRISMA flowchart is illustrated in Fig. 1.

### 3.2 Description of the included studies

Nineteen studies with 14,943 participants from 12 different countries (Australia, Bangladesh, Canada, China, France, Germany, Italy, Lebanon, Norway, Qatar, South Korea, and the U.S.) were included. Most studies (17 out of 19) were conducted in developed countries. All studies had female and male participants, with 53.68% being female overall. The smallest sample size was 72 (refugee subgroup from the U.S.), and the largest sample size was 5,925 (from Qatar). The mean age of participants was 44.84 years with an age range between 15 and 90 years. Almost all studies used a cross-sectional design, with one longitudinal study. Participant groups consisted of migrant populations in 10 studies; refugee populations in 5 studies; foreigner, Temporary Foreign Worker (each in one study) and undocumented immigrant populations in two studies. Table 1 provides the summary characteristics of all included studies.

**Table 1:** Summerized characteristics of included studies.

Author, Year	Host Country, Origin Country	Development status*	GDP** GDP per capita Rank**	COVID vaccination coverage# COVID mortality per 100,000 ##	Participants group Sample size	Data Collection method	Age Range / Mean	Contributing Factors	NOS total score
Salibi N et al., 2021 [47]	Lebanon, Syrian	Developing	NR	35 154.73	Refugee 1037	Online	50–90	Reasons for refusal were: newness of the vaccine; preference to maintain precaution measures; belief that the COVID-19 vaccine is not essential.	6
Sudhinaraset M et al., 2022 [48]	USA,	Developed	22939.58 2	67 314.76	Undocumented immigrant 326	Face to face		Gender, race/ethnicity, enrolled in school, health insurance	6
Lin S et al., 2022 [49]	Canada,	Developed	2027.37 15	84 113.5	Migrant 598	Online	25–65	Immigrant (vs non-immigrant) participants reported higher concerns regarding vaccine safety, adverse effects, mistrust in vaccinations	6
Thomas C.M et al., 2022 [50]	USA,	Developed	22939.58 2	67 314.76	Refugee 72	Face to face	18–70	Not reporting any vaccine concerns, male >=65, being a physician or advanced practice provider, interacting directly with patients from refugee, immigrant, and migrant communities.	5
Thomas C.M et al., 2022 [50]	USA,	Developed	22939.58 2	67 314.76	Migrant 195	Face to face	18–70		
Shaw J et al., 2022 [51]	USA, African	Developed	22939.58 2	67 314.76	Refugee 244	Face to face	18–65 (38.5)	Dd not observe any significant correlation between socio-demographic variables, country of origin, and vaccination status/intent.	6
West H et al., 2021 [52]	Bangladesh, Temporary Bangladesh Workers came back home	Developing	953.39 31	74 17.8	Temporary Foreign Workers 341	Face to face	40.6	Worried about getting COVID-19, location	5
Akintunde T.Y et al., 2022 [53]	China,	Developed	27071.96 1	90 1.05	Migrant 498	Online	15–44	Females, gender minorities, students, preference for alternative medicine, culture neutrality, belief against vaccination, and prefer free vaccination were less likely to pay for COVID-19 vaccination.	7
Diaz E et al., 2022 [54]	Norway, Bergen	Developed	445.51 4	76 70.72	Migrant 1284	Online	18–60	Duration of residence, gender, education	6
Achangwa C et al., 2021[55]	South Korea, Asians	Developed	2503.4 14	86 49.74	Foreigners 710	Online	25–35	Doctors’ recommendation , vaccine price, vaccine effectiveness, vaccine importance, vaccine safety	6
Alabdulla M et al., 2021 [56]	Qatar,	Developed	179.57 NR	97 23.64	Migrant 5925	Online	18–65	Citizens, females, concerns around the safety of COVID-19 vaccine and its longer-term adverse effects were main concerns cited.	6
Zhang M et al., 2021 [57]	USA,	Developed	22939.58 2	67 314.76	Refugee 435	Online	30–41	Being male, an essential worker	6
Wu L et al., 2022 [58]	China, Shanghai	Developed	27071.96 1	90 1.05	Migrant 530		>60 (71.3)	Gender, age, education, marital status	8
Aktürk Z et al., 2021[59]	Germany, Turkish	Developed	4843.39 5	76 175.05	Migrant 348		42.2	–	7
Ebrahimi OV et al.,2021 [60]	Norway,	Developed	445.51 4	76 70.72	Migrant 270	Online	18–86 (36.66)	Perceived risk of vaccination, belief in the superiority of natural immunity, fear concerning significant others being infected by the virus, and trust in health officials’ dissemination of vaccine-related information	6
Liddell BJ et al., 2021 [61]	Australia,	Developed	1427.26 19	86 227.31	Refugee 516	Online	20–80	Gender, information and trust barriers, lower logistical barriers, and attitudes	6
Carroll JK et al., 2021 [62]	USA, Hispanic/ Latin	Developed	22939.58 2	67 314.76	Migrant 325		49.2	African-American/Black and poorer reported physical health were associated with higher likelihoods of COVID-19 vaccine hesitancy.	5
Holz M et al., 2022 [63]	Germany, European	Developed	4843.39 5	76 175.05	Migrant 477	Online	41.56	European compared to Non-European migrant participants reported less political trust, more fear of personal infection and lower vaccination intentions.	6
Page KR et al., 2022 [64]	USA & Italy & France, Origin country not reported	Developed	Due to be a multi country study, this data could not be extracted		Refugee 812		38.7	Increasing age, the presence of comorbidities, and positive views about vaccination in general and COVID-19 in particular were all significantly associated with increased demand for vaccination, while living in France and relying on community network to get informed were associated with lower demand.	6

* Development status obtained from latest world bank data.

** GDP and GDP per capita rank obtained from https://statisticstimes.com/economy/projected-world-gdp-ranking.php

# COVID vaccination coverage obtained from https://www.nytimes.com/interactive/2021/world/covid-vaccinations-tracker.html

## COVID mortality per 100,000 obtained from https://coronavirus.jhu.edu/data/mortality

### 3.3 Methodological quality appraisal

**Fig. 2:**
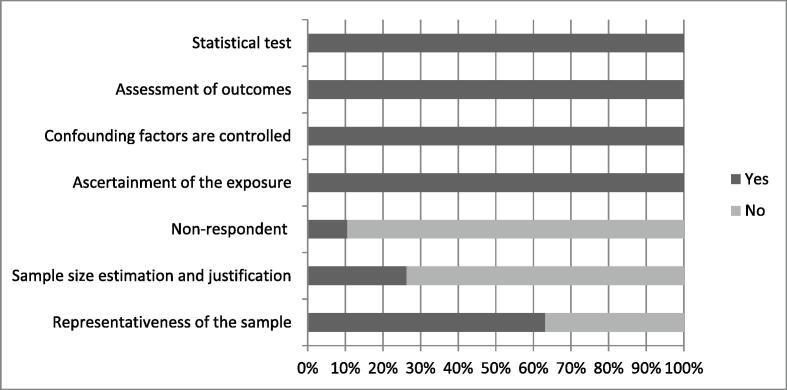
Details of the methodological quality appraisal of the included studies.

Most studies (15 out of 19) were categorized as being of high quality (or having low risk of bias). The total score of methodological quality is provided in Table 1, with details in Fig. 2. The main methodological problems were: (1) description of the response rate or the characteristics of the responders and the non-responders not having been reported in 17 out of 19 studies; (2) explanation regarding sample size estimation and justification not having been reported in 14 out of 19 studies; (3) a representative sample not having been recruited in 7 out of 19 studies (i.e., a selected group of a population was recruited or descriptions regarding the sampling strategy were not provided).

### 3.4 Estimation of pooled prevalence of *COVID-19 vaccine acceptance*

**Fig. 3:**
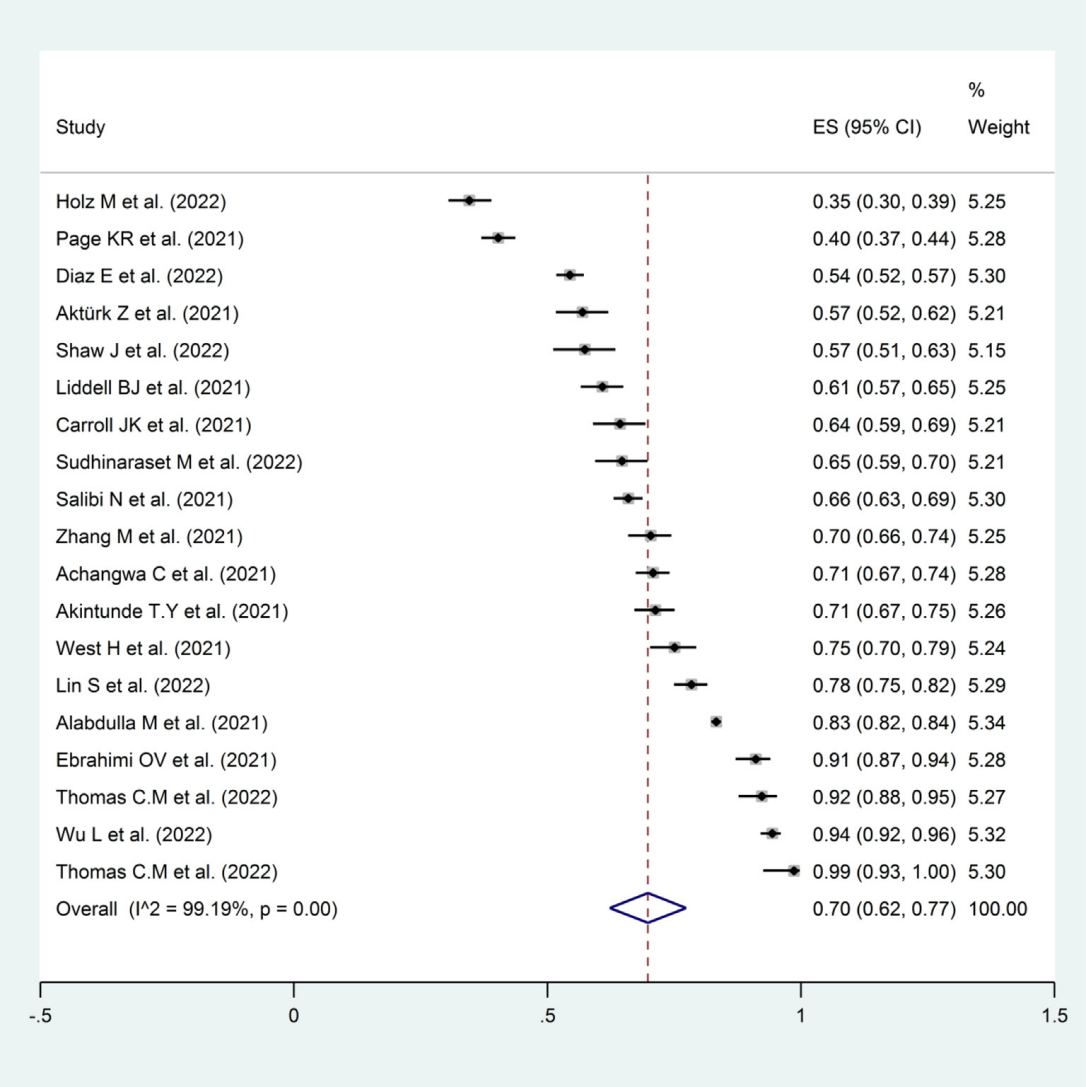
The forest plot of the pooled prevalence estimates of COVID-19 vaccine acceptance among migrant and refugee groups.

**Fig. 4:**
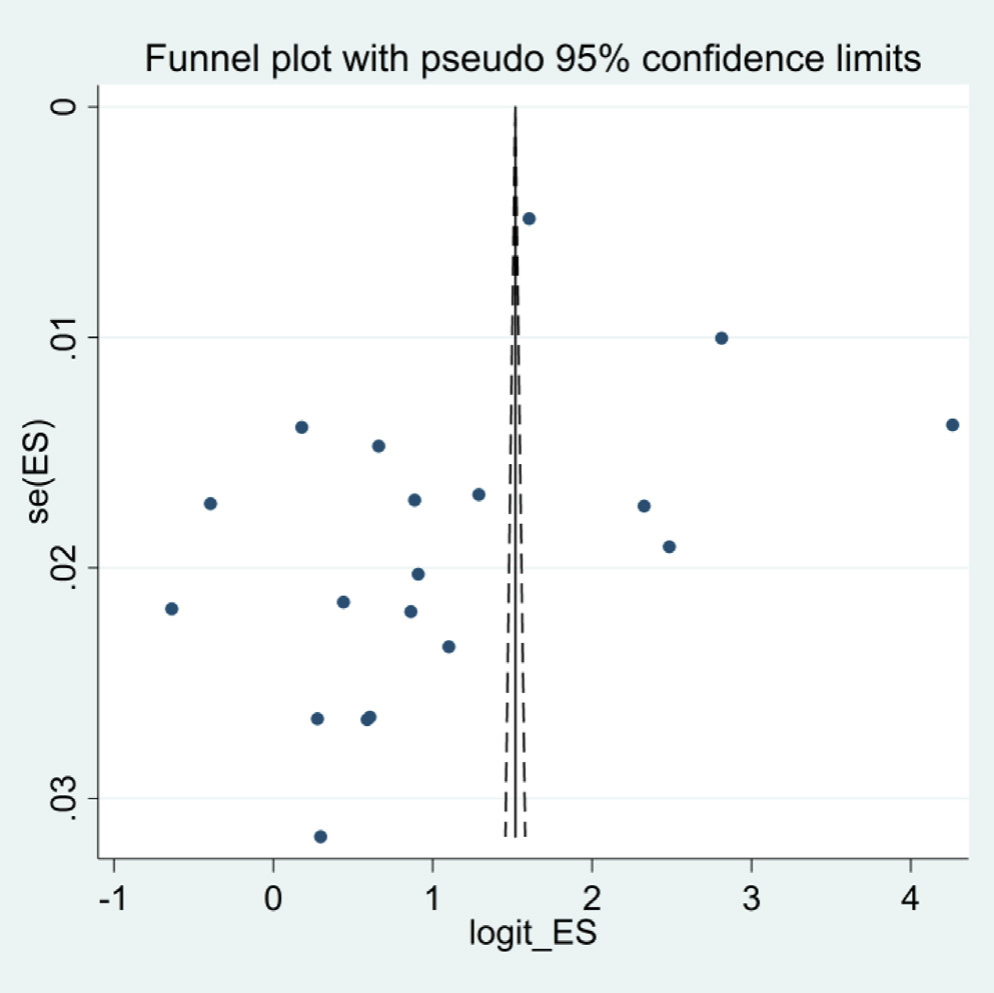
The funnel plot assessing publication bias among included studies reporting prevalence estimates of COVID-19 vaccine acceptance among migrant and refugee groups.

**Fig. 5:**
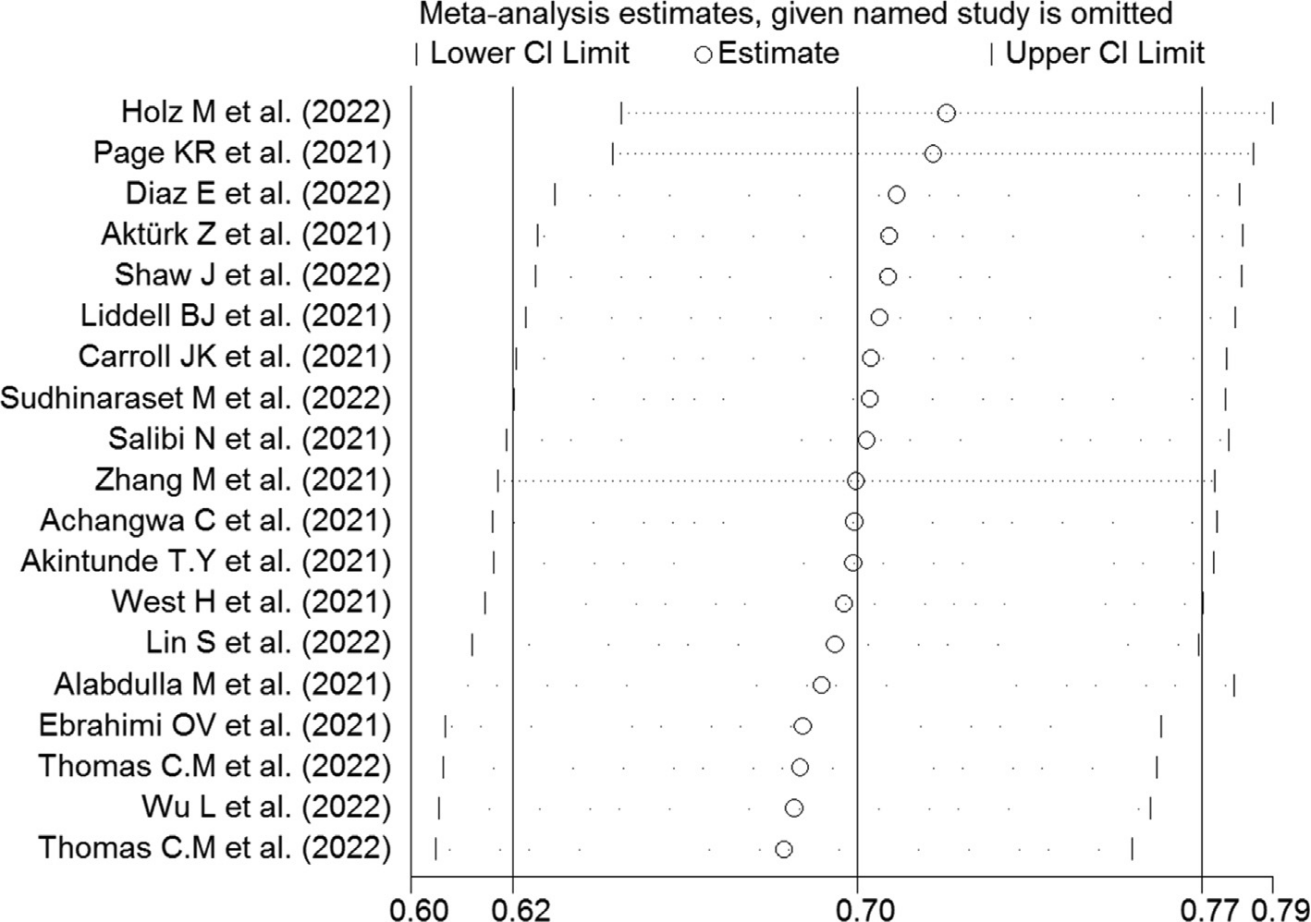
The sensitivity analysis plot assessing small study effects among included studies reporting prevalence estimates of COVID-19 vaccine acceptance among migrant and refugee groups.

The pooled estimated prevalence of COVID-19 vaccine acceptance was 70% (19 studies, 95% CI: 62.3–77.4%, I^2^: 99.19%, τ^2^: 0.03). Fig. 3 provides the forest plot regarding the pooled prevalence of COVID-19 vaccine acceptance among migrant and refugee groups. Gender-specific pooled prevalence estimates of COVID-19 vaccine acceptance were 59% (7 studies, 95% CI: 38–81%, I^2^: 99.41%, τ^2^: 0.08) for female participants and 60% (7 studies, 95% CI: 42–78%, I^2^: 98.97 %, τ^2^: 0.06) for male participants. Female and male participants did not differ significantly with each other (*p* = 0.64). The probability of publication bias was assessed and ruled out using Egger’s test (*p* = 0.15) and funnel plot approaches (Fig. 4). Sensitivity analysis (based on the one-out or Jack-knife method) showed that the pooled effect size was not affected by a single-study effect (Fig. 5).

Moderator variables of COVID-19 vaccine acceptance were assessed using univariable (Table 2) and multivariable (Table 3) *meta*-regression. Based on results of multivariable regression analysis, methodological quality (low vs. high risk of bias, p = 0.58), mean age of participants (p = 0.85), participant group (migrant, refugee, foreigners with p = 0.88) and country of origin (p = 0.50) were not significant moderators of COVID-19 vaccination acceptance among migrant and refugee, although the model explained 67% of variance.

**Table 2:** Results of uni-variable meta-regression regarding estimated pooled prevalence.

	**Variable**	**Number of studies**	**Coefficient**	**S.E.**	***p***	**I^2^ res. (%)**	**Adj. R^2^ (%)**	**τ^2^**
Host country characteristics	developmental status (developed vs. developing)	19	−0.008	0.13	0.95	99.21	−5.92	0.03
	GDP	17	3.62e−06	3.69e−06	0.34	99.01	−0.11	0.03
	GDP per capita Rank	15	−0.002	0.005	0.68	98.76	−6.24	0 .02
	COVID vaccination coverage	18	0.002	0.003	0.57	98.95	−4.09	0.03
	COVID mortality per 100,000	18	−0.0001	0.0003	0.60	99.03	−4.43	0.03
Origin country		9	0.04	0.02	0.03	98.52	45.43	0.02
Participant group (migrant, refugee, foreigners)		19	−0.035	0.03	0.26	99.04	2.14	0.03
Mean age of participants		8	0 .009	0.007	0.22	99.16	11.11	0.04
Methodological quality (low vs. high risk of bias)		19	−0.16	0.09	0.10	99.18	10.28

**Table 3:** Results of multivariable meta-regression regarding estimated pooled prevalence.

	**Coefficient**	**S.E.**	**p**	**Model Summary**
Methodological quality (low vs. high risk of bias)	−0.12	0.15	0.58	I2 res. (%) = 94.58%
Mean age of participants	0.003	0.01	0.85	Adj. R2 (%) = 66.99%
Participant group (migrant, refugee, foreigners)	−0.03	0.13	0.88	tau2 = 0.01
Origin country	0.05	0.05	0.50	Number of studies = 6

### 3.5 Contributing factors of COVID-19 vaccine acceptance

Contributing factors of COVID-19 vaccine acceptance included origin of migrants [59], [60], gender [28], [61], [62], [63], [64], [65], [66], [67] age [63], [65], [67], being enrolled in school [66] and being educated [28], [63], [67], marital status [63], durations of residence [28], [67], having health insurance [66], political mistrust [59], [61] and mistrust in vaccinations [65], [68], perceived individuals’ physical health [60], perceived risk of vaccination [69], [70], preference to maintain precaution measures, [71], the belief that COVID-19 vaccines are not essential [71], superiority of natural immunity [69], fear of personal infection [59], being worried about getting COVID-19 infection [72], fear concerning significant others being infected by the virus [69], trust in health officials’ dissemination of vaccine-related information [69], lower logistical barriers and attitudes [15], physicians’ recommendation, vaccine price, vaccine effectiveness and importance [70], concerns regarding newness of the vaccines [71], vaccine safety [62], [65], [68] and longer-term adverse effects [62], and being a physician or advanced practice provider interacting directly with patients from refugee, immigrant, and migrant communities [65].

## 4 Discussion

A main motivation of this review was the previous evidence of lower vaccination rates associated with higher burden of VPDs among migrant and refugee groups [21], [22], [27], [73]. Additionally, despite the need for timely data on COVID-19 vaccine acceptance and coverage among migrant and refugee populations, insufficient and scarce data currently exist. This situation highlighted the need for intensive and systematic research addressing this key topic [74]. In turn, the results of this review could help to devise well-informed and fine-tuned strategies to promote vaccine uptake among migrant and refugee groups, as well as to reveal the existing gaps in knowledge on this timely issue.

### 4.1 Pooled estimate of migrant COVID-19 vaccination acceptance compared to current evidence

The major finding of this review was the slightly higher rates of COVID-19 vaccine hesitancy among migrant/refugee groups compared to the latest estimates of COVID-19 vaccine acceptance in different categories worldwide [75]. Specifically, the current review showed that the willingness to get COVID-19 vaccines among migrant and refugee groups was 67%, while COVID-19 vaccine acceptance among the same groups was 73%. In a majority of included studies, the vaccine acceptance and intention to get vaccinated were generally close to the pooled estimate found in this review. An exception was a multi-national multicentric cross-sectional survey study among undocumented migrant groups in Geneva, Switzerland (54%), Baltimore, US (18%), Milan, Italy (16%) and Paris, France (13%) [76]. This study showed a low intention to get COVID-19 vaccination (41%) in undocumented migrant groups across different jurisdictions [76].

The most recent *meta*-analysis tackling the issue of COVID-19 vaccine hesitancy worldwide reported prevalence of COVID-19 vaccine acceptance at 75% [75]. However, the temporal, geographic and cultural variability in these estimates is an essential aspect that should be considered in the quest to interpret vaccine acceptance rates. This is attributed to the nature of vaccine hesitancy which is considered a time-, place-, and context-specific phenomenon [12]. As an example, an earlier *meta*-analysis reported a prevalence of COVID-19 vaccine acceptance at 61%, which is lower than the estimates observed in the current review [77]. Furthermore, our findings could not accurately depict the actual COVID-19 vaccine acceptance rates among refugee and migrant groups worldwide. This may be related to the relative scarcity of published reports that have assessed COVID-19 vaccine hesitancy and its associated determinants in countries hosting a large number of refugee people. Specifically, only 19 reports were eligible to be included in our review. A majority of the included studies originated from countries that are not ranked among the top 5 hosting countries for refugee groups, with Germany as the only exception (the others being Turkey, Colombia, Uganda and Pakistan) [78]. This highlights the need for more studies from low- and middle-income countries where a considerable fraction of the migrant/refugee populations are present (e.g. in Turkey, Iran and Jordan) [79].

### 4.2 Contributing factors to migrant COVID-19 vaccination acceptance

#### 4.2.1 Muultivariable model

The multivariable model did not identify any one factor that contributed at a statistically significant level. However, the model accounted for 67% of the variance. It is possible for a multivariable *meta*-regression model to account for a high proportion of the variance even when there are no significant moderators. This is because the model may still include important predictors that have a significant association with the outcome, but these predictors may not interact with the moderators in a way that is statistically significant. It is also possible that the lack of significant moderators is due to a lack of power or sample size to detect smaller effects. Additionally, it is possible that the moderators included in the model are not the most appropriate or relevant ones for the outcome of interest, and other potential moderators were not considered.

In summary, while the absence of significant moderators may be surprising, it does not mean that the model is flawed or unreliable. The interpretation of the results should take into account the limitations of the study design, sample size, and the potential for other unmeasured moderators. As such, we will discuss the individual variables below.

#### 4.2.2 Origin country

An important finding of the current review was the observation that COVID-19 vaccine acceptance rates were close to the estimates in countries of origin rather than the host countries with a few exceptions. For example, a study that involved Bangladeshi temporary foreign worker (TFW) group, the majority of whom were working in the Gulf Cooperation Council (GCC) countries of the Middle East where COVID-19 vaccine hesitancy is widely prevalent [8], [9], [72]. This longitudinal study showed rates for COVID-19 vaccine acceptance ranging between 83% in late December 2020/early January and 75% in February 2021 [72]. This result was slightly higher compared to the rates reported in Bangladesh and substantially higher compared to the vaccine acceptance rates reported in the host countries [8]. Another study from Qatar was unique in that it studied COVID-19 vaccine hesitancy among the general public residing in the country where 90% of the population are considered migrant [62]. This study found that the local Qatari population has a significantly lower intention to get vaccinated against COVID-19 (57%), compared to the migrant group (83%) [62]. Two studies that were conducted in Germany showed a similar pattern as well. The study by Holz et al. involved 477 individuals considered migrant, with a majority of European origin, and revealed that the intention for COVID-19 vaccination was higher among native Germans [59]. Another study was conducted in Munich, Germany among 420 Turkish- and German-speaking patients of Turkish-speaking family doctors in Munich [80]. The intention to receive COVID-19 vaccination was 48%, which is lower than the figures reported in Turkey and much lower than those reported among health professionals in Germany [81], [82]. A study conducted in different regions of the U.S. involved 435 people of refugee status and showed a wide variability in COVID-19 vaccine acceptance by country of origin as follows: Bhutan (78%), Afghanistan (71%), Somalia (66%), Myanmar (65%), and South Sudan (62%) [64]. These estimates were closer to those reported in countries of origin in contrast to those reported in the U.S. [8], [64]. Similarly, the vaccine acceptance rates in another study from the U.S. were largely a reflection of COVID-19 vaccine acceptance rates in the regions of participants’ origin as follows: North Africa (42%), Middle East (46%), East/Southeast Asia (53%), East Africa (54%), Central Africa (60%), Latin America (74%), and South Asia (91%) [8], [67].

An Australian study was an exception to the previously mentioned pattern. This study by Liddell et al. involved 1085 people of refugee status who arrived into Australia since 2011, the majority of whom had secured a visa [61], [83]. This cohort involved a majority (85%) of participants coming from three Middle Eastern countries (Iraq, Iran and Syria) [61]. Hesitancy to COVID-19 vaccination was reported in 24% of participants. This rate was closer to COVID-19 vaccine hesitancy rates reported in Australia rather than countries of origin for most participants [8], [84]. Speculatively, the vaccine hesitancy rates reported may be attributed to living in the country for longer periods with subsequent adoption of the prevalent attitudes in the host country, and this possibility warrants direct testing.

A possible explanation for the aforementioned results may also be related to integration difficulties faced by refugee groups in host countries including marginalization from healthcare systems [85], [86]. This issue was manifested in the studies among refugee groups in Norway, where one large study from the country involved 1284 immigrant individuals in Oslo. The results revealed that people of migrant status had a lower likelihood of being offered to get vaccinated compared to native Norwegians [28]. This comes in spite of similar rates of COVID-19 vaccine hesitancy in people of migrant status and native citizens as showed in a recent study by Ebrahimi et al. in the Nordic country [69].

#### 4.2.3 Other potentially contributing factors

Similar to what has been reported in the studies among general populations worldwide [9], [75], the factors associated with COVID-19 vaccine hesitancy commonly found in this review included: sociodemographic variables [61], [62], [63], [64], [65], [66], [67], low confidence in vaccine safety and fear of adverse effects [62], [65], [68], [70], and high complacency [59], [60], [71]. Additionally, the negative attitude towards compulsory vaccination (i.e. “being forced to get the COVID-19 vaccine”) was also linked to higher rates of vaccination hesitancy [61].

For example, a study among adult foreign populations in South Korea showed that concerns about adverse effects of COVID-19 vaccination was the most common factor associated with vaccine hesitancy [70]. In the US-based study that involved people of refugee status in 32 states besides Washington D.C., COVID-19 vaccine hesitancy was associated with concerns regarding vaccine safety as well [64]. Perceived vaccine safety was an important determinant of COVID-19 vaccine acceptance in a study among older adults by Wu et al. in Shanghai, China, including 530 people of migrant status who participated in the study [63]. In China, another study by Akintunde et al. among 498 people of migrant status (students, expatriates, and business owners) investigated the willingness to pay for COVID-19 vaccination [87]. This study showed the importance of perceived vaccine efficacy and perceived severity of the disease in willingness to pay for the vaccine.

Fear of adverse effects and lower levels of perceived safety of vaccination could be linked to higher rates of COVID-19 vaccine hesitancy among females [9], [88], [89]. This pattern was reported in various studies addressing the determinants of vaccination hesitancy [20], [75], [77], [90]. In this review, several included studies revealed a similar result [62], [64], [65]. Thus, the focus on females in the efforts to promote vaccination may warrant special attention to build trust regarding the importance and safety of the currently available vaccines. Nevertheless, other studies revealed the opposite pattern and overall no gender-related differences were observed, suggesting the need for more studies to evaluate the role of gender in relation to other factors in determining intentions to get vaccinated [66], [76].

Racial background was another factor linked to COVID-19 vaccine hesitancy among migrant groups as indicated in a study that involved Asian and Latino participants in California, U.S. [66]. This study showed a higher vaccine acceptance among Latino compared to Asian participants. Furthermore, the study showed that immigration enforcement exposure was linked to COVID-19 vaccine hesitancy, adding more evidence to the literature pointing to the negative impact of the immigration enforcement on health-seeking behavior [91].

Another factor associated with COVID-19 vaccine hesitancy was reported by Salibi et al. which involved people of refugee status aged 50 years or older from Syria and in Lebanon [71]. The results pointed to COVID-19 vaccine hesitancy rate of 34%, even if the vaccine were safe and free. Notably, vaccine hesitancy was more prevalent among refugee groups outside versus inside informal tented settlements [71].

To summarize, the aforementioned results highlight the importance of delivering proper messages emphasizing the safety of vaccination and its efficacy, besides the need for emphasis on the potential severity of COVID-19 which in turn could help to lower complacency levels. Conditions of living and access to healthcare systems are other factors that should be considered in efforts aiming to promote vaccine acceptance and uptake among people of migrant and refugee status.

### 4.3 Limitations

The results of this study can be highly valuable; however, it should be interpreted in light of several limitations as follows. (1) As mentioned earlier, the phenomenon of COVID-19 vaccine hesitancy may be time-, place- and context-specific. A vast majority of studies included in this systematic review were cross-sectional, with variable timing of the surveys during the pandemic. The timing of surveys in relation to different waves of COVID-19 in different countries may have affected the self-reported willingness to get vaccinated in different study participants, which should be considered in efforts aimed at interpreting the results of these studies. (2) The estimation of COVID-19 vaccine hesitancy was based on different survey instruments and item phrasings. Therefore, this approach could have contributed to differences in COVID-19 vaccine hesitancy estimates. (3) There is a risk of selection bias based on the observation that a considerable fraction of the included studies were conducted in the US. Further, sample size and sampling approach (online, face-to-face) varied across different studies, and a lower number of studies were conducted among refugee groups. (4) While we did not find gender-related differences in COVID-19 vaccine acceptance (including according to host country characteristics), we note that vaccination acceptance was defined mostly based on origin countries of minority groups. Different cultures of origin and host countries may have important influences. As the total number of included studies was low (19 manuscripts) and the number of studies reporting origin countries of migrant was much lower (6 manuscripts), further analysis to understand potential cultural differences was not possible in the present systematic review. Cultural differences and how they may relate to gender/sex should be investigated further and reported in future studies. Also of note, only six studies were included in the multivariate *meta*-regression model, which may limit the statistical power to identify significant moderators. Because of this, the confidence intervals were wide around the estimated coefficients, making it difficult to detect statistically significant effects. Given this limitation, it is important to interpret the results with caution and to consider the potential for unmeasured moderators that could influence the relationship between predictors and outcome. Future research with larger sample sizes and more studies could help improve the statistical power and generalizability of the findings.

## 5 Conclusions

The rates of COVID-19 vaccine hesitancy among migrant and refugee groups were consistent with the latest estimates worldwide. The notion that migrant or refugee groups have higher rates of COVID-19 vaccine hesitancy was not supported by this review. Analyses of the factors associated with COVID-19 vaccine hesitancy were largely consistent with what has been reported in different studies among the general public worldwide. Vaccine safety and efficacy appeared among the most important determinants of COVID-19 vaccine acceptance. Therefore, effective communication and educational programs emphasizing vaccine safety, efficacy, and absence of long-term adverse events may be helpful to increase COVID-19 vaccine coverage among migrant and refugee populations. We recommend further studies to evaluate the attitude and vaccine coverage in countries hosting large numbers of people of refugee/migrant status based on the observation of limited literature focusing on this timely and important issue.

## Consent to participate

Not applicable.

## Consent to publish

Not applicable.

## Funding

None.

## Declaration of Competing Interest

The authors declare that they have no known competing financial interests or personal relationships that could have appeared to influence the work reported in this paper.

## Data Availability

Data will be made available on request.
